# Rapid and sensitive detection of canine distemper virus by real-time reverse transcription recombinase polymerase amplification

**DOI:** 10.1186/s12917-017-1180-7

**Published:** 2017-08-15

**Authors:** Jianchang Wang, Jinfeng Wang, Ruiwen Li, Libing Liu, Wanzhe Yuan

**Affiliations:** 1 Center of Inspection and Quarantine, Hebei Entry-Exit Inspection and Quarantine Bureau, No.318 Hepingxilu Road, Shijiazhuang, Hebei Province 050051 People’s Republic of China; 2 College of Veterinary Medicine, Agricultural University of Hebei, No.38 Lingyusi Street, Baoding, Hebei 071001 People’s Republic of China

**Keywords:** Canine distemper virus, Nucleocapsid protein gene, Exo probe, Recombinase polymerase amplification, RPA and CDV

## Abstract

**Background:**

Canine distemper, caused by Canine distemper virus (CDV), is a highly contagious and fatal systemic disease in free-living and captive carnivores worldwide. Recombinase polymerase amplification (RPA), as an isothermal gene amplification technique, has been explored for the molecular detection of diverse pathogens.

**Methods:**

A real-time reverse transcription RPA (RT-RPA) assay for the detection of canine distemper virus (CDV) using primers and exo probe targeting the CDV nucleocapsid protein gene was developed. A series of other viruses were tested by the RT-RPA.Thirty-two field samples were further tested by RT-RPA, and the resuts were compared with those obtained by the real-time RT-PCR.

**Results:**

The RT-RPA assay was performed successfully at 40 °C, and the results were obtained within 3 min–12 min. The assay could detect CDV, but did not show cross-detection of canine parvovirus-2 (CPV-2), canine coronavirus (CCoV), canine parainfluenza virus (CPIV), pseudorabies virus (PRV) or Newcastle disease virus (NDV), demonstrating high specificity. The analytical sensitivity of RT-RPA was 31.8 copies in vitro transcribed CDV RNA, which is 10 times lower than the real-time RT-PCR. The assay performance was validated by testing 32 field samples and compared to real-time RT-PCR. The results indicated an excellent correlation between RT-RPA and a reference real-time RT-PCR method. Both assays provided the same results, and R^2^ value of the positive results was 0.947.

**Conclusions:**

The results demonstrated that the RT-RPA assay offers an alternative tool for simple, rapid, and reliable detection of CDV both in the laboratory and point-of-care facility, especially in the resource-limited settings.

## Background

Canine distemper, caused by canine distemper virus (CDV), is a highly contagious and fatal systemic disease found worldwide not only in dogs and many other carnivores but also in some non-carnivores [1]. CDV is a non-segmented, negative-stranded, enveloped RNA virus that belongs to the family *Paramyxoviridae* and the genus *Morbillivirus*, and is one of the most lethal infectious agents in both susceptible free-living and captive carnivores [2]. CDV-infected dogs may develop respiratory, gastrointestinal, dermatologic, ophthalmic or neurological disorders, that appear simultaneously or sequentially [3, 4]. The broad spectrum of clinical signs, not dissimilar from the signs observed in other respiratory and enteric diseases of dogs, hampers accurate and early clinical diagnosis of canine distemper [5]. Therefore, rapid and accurate diagnosis of CDV infection would enable veterinarians to implement appropriate strategies in time to improve disease management and prevent outbreaks, particularly within a shelter environment.

With the advances in molecular detection techniques, a substantial number of assays have been described for CDV diagnosis with a varying degree of sensitivity and specificity, such as reverse transcription polymerase chain reaction (RT-PCR) [6], nested RT-PCR [7], real-time RT-PCR [8], reverse transcription loop-mediated isothermal amplification (RT-LAMP) [9] and insulated isothermal PCR (iiPCR) [1]. The RT-PCR assays however, are cold chain dependent and require relatively expensive equipment with experienced technicians, making these assays difficult to implement in the field and at the point-of-care. Recently, an iiPCR method was reported for rapid and sensitive detection of CDV [1], but the reaction time was about 1 h. A simple, rapid, accurate and user-friendly platform is still needed for early point-of-care (POC) detection of CDV infection.

Recombinase polymerase amplification (RPA) is an isothermal gene amplification technique that has been demonstrated to be a rapid, specific, sensitive, and cost-effective molecular method to identify pathogens [10, 11]. As with PCR, the use of two opposing primers allows exponential amplification of the target sequence in RPA, but the latter is tolerant to 5–9 mismatches in primer and probe showing no influence on the performance of the assay [12–14]. It is thought that RPA possesses superiority in speed, portability and accessibility over PCR [15]. Indeed, RPA has recently been explored to replace PCR for the molecular detection of diverse pathogens with different detection strategies, e.g., bacteria, fungi, parasites and viruses [13, 15–21]. In this study, the development of a real-time RT-RPA assay for simple, rapid, portable and POC detection of CDV was described. The fluorescence in the assay is produced by an exo probe which is detected with a portable, user-friendly tube scanner (Genie III, OptiGene Limited, West Sussex, United Kingdom). The Genie III used in the study weighs only 1.75 kg, measuring 25 cm × 16.5 cm × 8.5 cm, and incorporates a rechargeable battery that can support operation for a whole day, making it suitable for point-of-care testing.

## Methods

### Virus strains and clinical samples

Canine distemper virus (CDV-FOX-TA strain, genotype: America-2) [22], canine parvovirus (CPV, CPV-b114 strain), canine coronavirus (CCoV, ATCC VR-809 strain), canine parainfluenza virus (CPIV, CPIV/A-20/8 strain), pseudorabies virus (PRV, Barth-K61 strain) were maintained in our laboratory. Newcastle disease virus (NDV, LaSota strain) was from the commercial live vaccine (Weike Biotechnology, Harbin, China). Thirty-two nasal/oropharyngeal swabs were collected from 20 dogs of both sexes (various breeds and ages) from the animal hospital of Agricultural University of Hebei and 12 raccoon dogs from the farms in Hebei Province, China from 2014 to 2016 and snap-frozen for storage at −80 °C. All the dogs and raccoon dogs clinically were suspected of being CDV infected. Fifteen samples from the dogs and 5 samples from the raccoon dogs had been tested to be CDV positive with the Ct values ranging from 16.36 to 37.03, and the other 12 samples were CDV negative by the real-time RT-PCR [8].

### DNA/RNA extraction

CDV, CCoV, CPIV and NDV viral RNA was extracted using Trizol Reagent (Invitrogen, Waltham, USA) according to manufacturer’s instructions. CPV and PRV viral DNA was extracted using the TIANamp Virus DNA kit (Tiangen, Beijing, China) according to manufacturer’s instructions. Viral DNA and RNA were quantified using a ND-2000c spectrophotometer (NanoDrop, Wilmington, USA). For viral RNA extraction from the nasal/oropharyngeal swabs, the swab was inoculated and vortexed in 1 mL sterile phosphate-buffered saline and centrifuged at 10000 rpm for 10 min at 4 °C. The supernatant was collected and used for viral RNA extraction using the Trizol Reagent. Viral RNA extracted from clinical samples was finally eluted in 20 μL of nuclease-free water. All RNA and DNA templates were stored at −80 °C until tested.

### Generation of standard RNA

The 1572 bp RT-PCR product, which covers the nucleocapsid protein gene of CDV, was generated from viral genomic RNA template extracted from CDV-FOX-TA strain using N-forward and N-Reverse primers (Table 1). Primers were synthesized by Sangon (Sangon, Shanghai, China). The 50 μL reaction mixture consisted of 25 μL of 2 × 1 Step Buffer, 2 μL of 1 Step Enzyme Mix (Takara, Dalian, China), 0.5 μL of N-forward primer (20 μmol/L), 0.5 μL of N-Reverse primer (20 μmol/L), 5 μL of extracted RNA template and 17 μL of ddH_2_O. The reaction condition was set as follows: 50 °C for 30 min; 94 °C for 2 min; 32 cycles of 94 °C for 30 s, 55 °C for 30 s and 72 °C for 60 s. The resulting fragment was purified with the Gel Extraction Kit (Tiangen, Beijing, China), ligated into a pGEM-T Easy vector (Promega, Madison, WI, USA) and transformed into E.coli DH5α chemically competent cells (Dingguo, Beijing, China) according to standard procedures. Positive clones were identified by sequencing analysis. The recombinant plasmid DNA was linearized by Nde I (Takara, Dalian, China), purified using the Wizard SV Gel and PCR Clean-Up System (Promega, Madison, USA) and transcribed in vitro with the RiboMAX Large Scale RNA Production System-T7 (Promega, Madison, USA). In vitro transcribed CDV RNA was digested with the supplied RQ1 RNase-free DNase and purified. It is 97 nucleotides from the T7 promoter region to the NdeI cut site of the pGEM-T Easy vector. All transcripts generated were about 1669 nucleotides in length and approximate size and RNA integrity were verified by agarose gel electrophoresis. The in vitro transcripts were quantified using a ND-2000c spectrophotometer (NanoDrop, Wilmington, USA), and the copy number of RNA molecules was calculated by the following formula [23]: Amount (copies/μL = [RNA concentration (g/μL) /(transcript length in nucleotides × 340) × 6.02 × 10^23^.

**Table 1:** Sequence of primers and probes for CDV RT-PCR, real-time RT-PCR and RT-RPA assays

Name	Sequence 5′-3′	Amplicon size (bp)
N-Forward	ATGGCCAGCCTTCTTAAG	1572
N-Reverse	TTAATTGAGTAGCTCTCTATCA	
CDV-F	AGCTAGTTTCATCTTAACTATCAAATT	87
CDV-R	TTAACTCTCCAGAAAACTCATGC	
CDV-P	FAM-ACCCAAGAGCCGGATACATAGTTTCAATGC-BHQ1	
CDV-RPA-F	GCTTACTTCAGACTCGGGCAAGAAATGGTTA	154
CDV-RPA-R	CAGTAGCTCGAATTGTCCGGTCCTCTGTTGT	
CDV-RPA-P	CTTGGCATCACCAAGGAGGAAGCTCAGCTGG(FAM-dT) (THF)(BHQ1- dT)CAGAAATAGCATCCA-C3spacer	

### RPA primers and exo probe

Nucleotide sequence data for CDV strains from GenBank were aligned to identify regions that are conserved in the nucleocapsid gene. According to the reference sequences of different CDV genotypes (accession numbers: AB490678, AF164967, AY386316, GU138403, HQ540292, KF856711, KF914669), three forward primers, three reverse primers, and two exo probes were designed. The RPA primers and probes were tested to select the combination yielding the highest sensitivity (Table 1). Primers and exo probe were synthesized by Sangon (Sangon, Shanghai, China).

### Rt-Rpa

RT-RPA reactions were performed in a 50 μL volume using a TwistAmp™ RT exo kit (TwistDX, Cambridge, UK). Other components included 420 nM each RPA primer, 120 nM exo probe, 14 mM magnesium acetate, and 1 μL of viral or sample RNA. All reagents except for the viral template and magnesium acetate were prepared in a master mix, which was distributed into each 0.2 mL freeze-dried reaction tube containing a dried enzyme pellet. One μL of viral RNA was added to the tubes. Subsequently, magnesium acetate was pipetted into the tube lids, then the lids were closed carefully, the magnesium acetate was centrifuged into the rehydrated material using a minispin centrifuge. The sample was vortexed briefly and spun down once again, and the tubes were immediately placed in the Genie III scanner device to start the reaction at 40 °C for 20 min. The fluorescence signal was collected in real-time and would increase markedly due to successful amplification.

### Real-time RT-PCR

Real-time RT-PCR specific for CDV was performed on the ABI 7500 instrument as described previously with some modifications [8]. Sequences for the primers and probe are provided in Table 1. The One Step PrimeScript^®^ RT-PCR Kit (Takara Co., Ltd., Dalian, China) was applied in real-time PCR and the reaction was performed as follows: 42 °C 5 min; 95 °C for 10 s; then 40 cycles of 95 °C for 5 s and 60 °C for 35 s.

### Analytical specificity and sensitivity analysis

Ten ng of RNA or DNA was used as template for the specificity analysis of the RT-RPA assay. The assay was evaluated against a panel of pathogens considered important in dogs, CDV, CPV, CCoV, CPIV, PRV, and the virus also belonging to the family Paramyxoviridae, NDV.

The in vitro transcribed RNA was diluted in a 10-fold serial dilutions to achieve RNA concentrations ranging from 9.4 × 10^5^ to 9.4 × 10^−1^ copies/μL, which were used as the standard RNA for CDV RT-RPA sensitivity assay. The RT-RPA was tested using the quantitative RNA in eight different times (inter-assay) and in eight replicates in one time (intra-assay) to determine the coefficient of variation (CV). The intra- and inter-assay CVs for the threshold times were calculated. The threshold time in eight different times was plotted against the molecules detected,and a semi-log regression was calculated using Prism software 5.0 (Graphpad Software, SanDiego, USA). For exact determination, a probit regression was performed using the Statistical Product and Service Solutions software (IBM, Armonk, USA).

### Validation with clinical samples

RNA extracted from 32 clinical swab samples was tested by RT-RPA, and the results were compared with those obtained using real-time RT-PCR [8].

## Results

### Analytical specificity and sensitivity

**Fig. 1:**
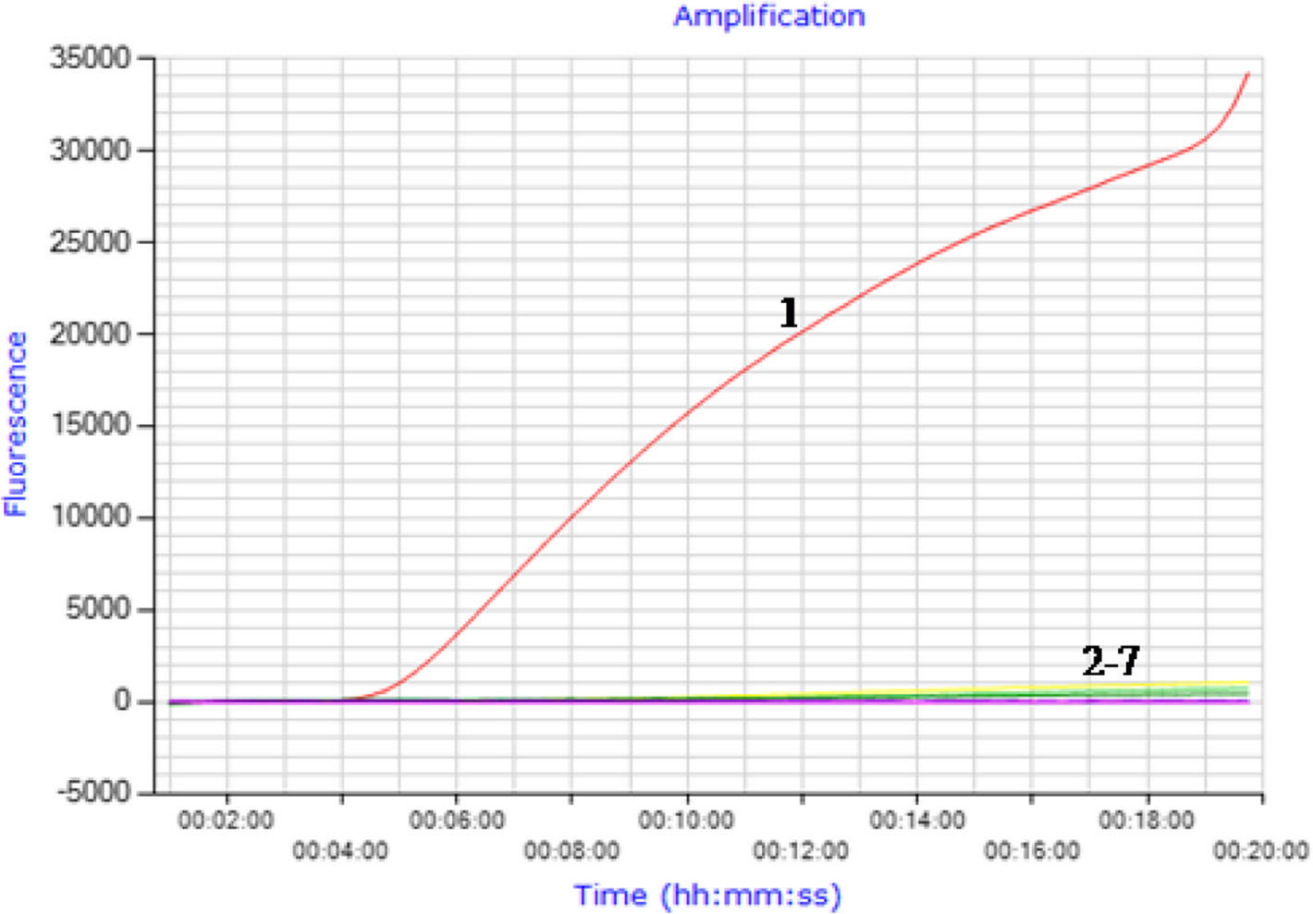
Analytical specificity of the CDV RT-RPA assay. RT-RPA was carried out at 40 °C for 20 min using 10 ng of viral RNA or DNA as template. The results showed RT-RPA amplified the CDV RNA, but not other viruses tested. 1, CDV; 2, CPV-2; 3, CCoV; 4, CPIV; 5, NDV; 6, PRV; 7, canine genome DNA

Using 10 ng of viral RNA, DNA or canine genome as template, the results showed that only the CDV was detected by RT-RPA while the other viruses and canine genome templates were not detected (Fig. 1, *n* = 5). No cross detections were observed. The data demonstrated the specificity of RT-RPA assay for the detection of CDV.

**Fig. 2:**
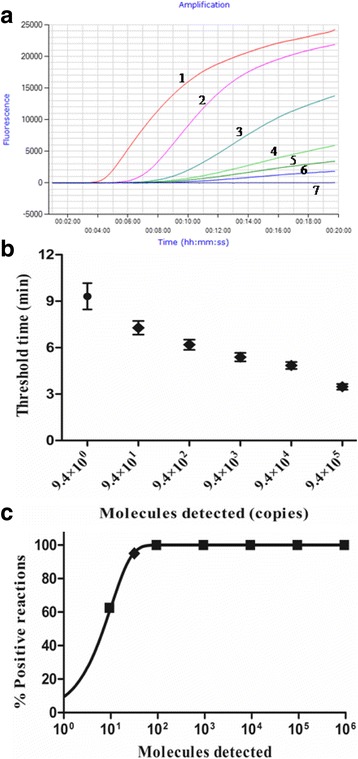
Performance of the CDV RT-RPA assay. **a** Fluorescence development over time using a dilution range of 9.4 × 10^5^ to 9.4 × 10^−1^ copies of the CDV standard RNA. Numbers for amplification curves were designated as, 1: 9.4 × 10^5^ copies; 2: 9.4 × 10^4^ copies; 3: 9.4 × 10^3^ copies; 4: 9.4 × 10^2^ copies; 5: 9.4 × 10^1^ copies; 6: 9.4 × 10^0^ copies; 7: 9.4 × 10^−1^ copies. **b** Semi-logarithmic regression of the data collected from eight CDV RT-RPA tests on the RNA standards using Prism Software 5.0. The run time of the RT-RPA was between 3 min–12 min for CDV RNA from 9.4 × 10^5^ to 9.4 × 10^0^ copies. **c** Probit regression analysis using SPSS software on data of eight runs. The detection limit at 95% probability (31.8 molecules) is depicted by a rhomboid

Using a dilution range of 9.4 × 10^5^ to 9.4 × 10^−1^ copies/μL of in vitro transcribed standard RNA as template, the RT-RPA and real-time RT-PCR were performed. As shown in Fig. 2, the detection limit of the RT-RPA was 9.4 copies (Fig. 2a and b), which was ten times lower than the real-time RT-PCR (data not shown). The RT-RPA assay was performed eight times on the quantitative RNA, in which 9.4 × 10^5^ to 9.4 × 10^1^ RNA molecules were detected in 8/8 runs, 9.4 × 10^1^, 5/8 and 9.4 × 10^−1^, 0/8 (Fig. 2b). Due to the inconsistency in the results, a probit regression analysis was applied, in which the sensitivity in 95% of cases was determined at 31.8 RNA molecules (Fig. 2c). With the data of eight runs on the quantitative RNA standards, a semi-log regression analysis showed the runtime of RT-RPA assay was approximately 3 min–12 min for 9.4 × 10^5^ to 9.4 × 10^0^ copies (Fig. 2b), while the Ct values of the real-time RT-PCR were 24.43–37.81, which needs approximately 36 min- 57 min.

For the 9.4 × 10^5^ to 9.4 × 10^0^ RNA molecules in the RT-RPA, the CV of the threshold time in the intra-assay ranged from 1.8% to 6.88%, while the CV in the inter-assay ranged from 3.74% to 10.61%.

### Evaluation of RT-RPA with clinical samples

**Fig. 3:**
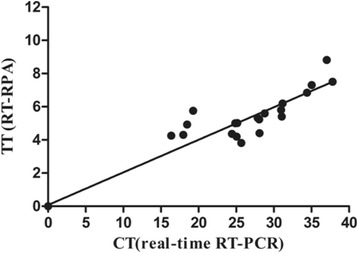
Comparison between performances of RT-RPA and real-time RT-PCR on clinical samples. Thirty-two RNA extracts of the clinical samples were screened. Linear regression analysis of RT-RPA threshold time (TT) values (y axis) and real-time RT-PCR cycle threshold (Ct) values (x axis) were determined by Prism software. R^2^ value was 0.947

The detection results of 32 clinical samples demonstrated that the RT-RPA and real-time RT-PCR showed the same performance (20 positive and 12 negative cases). The further analysis demonstrated the RT-RPA had a diagnostic agreement of 100% with real-time RT-PCR (Table 2). No discrepancy was found in samples (3/20) containing low levels of CDV RNA (Ct > 35, real time RT-PCR), indicating that the established RT-RPA reliably detected low amounts of CDV in clinical samples. Positive samples had real-time RT-PCR Ct values ranging from 16.36 to 37.03, indicating that the RT-RPA was able to detect CDV RNA across the entire range of the assay. The threshold time (TT) and cycle threshold (Ct) values of RT-RPA and RT-PCR were respectively well at an R^2^ value of 0.947 (Fig. 3).

**Table 2:** Detection of CDV in clinical samples by RT-RPA and real-time RT-PCR

		real-time RT-PCR		
		Positive	Negative	Total
RT-RPA	Positive	20	0	20
	Negative	0	12	12
	Total	20	12	32

## Discussion

In this study, we developed a RT-RPA method based on exo probe for the rapid and sensitive detection of CDV. Specificity analysis revealed that the RT-RPA assay could only detect the CDV, but not other viruses (Fig. 1). Other CDV genotypes were not included in the assay except for the genotype America-2, which is deficiency of the study. The RPA is tolerant to 5–9 mismatches in primer and probe showing no influence on the performance of the assay [12–14], and there were only 2–4 mismatches in the primers and probe in this study with other CDV genotypes. It is assumed the assay would detect all genotypes of CDV, based on targeting a conserved region, but this was not confirmed by testing validated genotypes. The detection limit of the RT-RPA was 9.4 copies, which was 10 times lower than the real-time RT-PCR described previously [8]. We further evaluated this method using clinical samples, and the diagnosis agreement of the RT-RPA and real-time RT-PCR was 100%. Interpretations were limited by the small sample-size, but the results suggested that the developed RT-RPA performed well in CDV detection. RT-RPA assay should be further tested to more CDV strain RNA extracts or clinical samples from various regions worldwide to evaluate sensitivity of the assay for detecting various strains of the virus circulating.

In recent years, a number of isothermal DNA amplification methods have been developed as a simple, rapid alternative to PCR-based amplification. In the RT-LAMP assay for CDV, four primers were needed and the optimal reaction condition was 60 min at 65 °C [9]. The developed iiPCR could detect as low as 7.6 copies of CDV RNA in approximately 1 h [1]. For the RT-RPA assay developed in this paper, it could detect 9.4 copies of CDV RNA in 12 min, which was more rapid than the above assays. Compared to other isothermal amplification techniques, RPA requires no initial heating for DNA denaturation, and the results could be obtained in less than 20 min; RPA demonstrates a certain tolerance to common PCR inhibitors, and could tolerate a wide range of biological samples [11]; RPA reagents in the lyophilized pellet form could be delivered and stored without cold chain, which could perform satisfactory at 25 °C for up to 12 weeks and at 45 °C for up to 3 weeks [24]. Thus, RPA may be the most applicable approach for the field and point-of-care diagnosis of infectious diseases [11]. A noteworthy feature of the developed RT-RPA assay, i.e., the use of the tube scanner Genie III makes on-site CDV detection feasible, which is especially important for CDV detection and epidemiological surveillance in the field.

## Conclusions

An RT-RPA assay with high analytical sensitivity and specificity was successfully developed for the rapid detection of CDV, which could be completed within 20 min. More importantly, the portable feature of the RT-RPA assay makes it applicable at quarantine stations, ports or the site of outbreak. The rapid, sensitive and feasible RT-RPA assay would be a useful tool in CDV control, especially in the resource-limited settings.

## Abbreviations

CCoV: Canine coronavirus
CDV: Canine distemper virus
CPIV: Canine parainfluenza virus
CPV-2: Canine parvovirus-2
CT: Cycle threshold
CV: Coefficient of variation
NDV: Newcastle disease virus
POC: Point-of-care
PRV: Pseudorabies virus
RPA: Recombinase polymerase amplification
TT: Threshold time

## References

[1] Wilkes RP, Tsai YL, Lee PY, Lee FC, Chang HF, Wang HT (2014). Rapid and sensitive detection of canine distemper virus by one-tube reverse transcription-insulated isothermal polymerase chain reaction. BMC Vet Res.

[2] Lednicky JA, Dubach J, Kinsel MJ, Meehan TP, Bocchetta M, Hungerford LL, Sarich NA, Witecki KE, Braid MD, Pedrak C (2004). Genetically distant American canine distemper virus lineages have recently caused epizootics with somewhat different characteristics in raccoons living around a large suburban zoo in the USA. Virol J.

[3] Beineke A, Puff C, Seehusen F, Baumgartner W (2009). Pathogenesis and immunopathology of systemic and nervous canine distemper. Vet Immunol Immunopathol.

[4] Tan B, Wen YJ, Wang FX, Zhang SQ, Wang XD, Hu JX, Shi XC, Yang BC, Chen LZ, Cheng SP (2011). Pathogenesis and phylogenetic analyses of canine distemper virus strain ZJ7 isolate from domestic dogs in China. Virol J.

[5] Seki F, Ono N, Yamaguchi R, Yanagi Y (2003). Efficient isolation of wild strains of canine distemper virus in Vero cells expressing canine SLAM (CD150) and their adaptability to marmoset B95a cells. J Virol.

[6] Frisk AL, Konig M, Moritz A, Baumgartner W (1999). Detection of canine distemper virus nucleoprotein RNA by reverse transcription-PCR using serum, whole blood, and cerebrospinal fluid from dogs with distemper. J Clin Microbiol.

[7] Shin YJ, Cho KO, Cho HS, Kang SK, Kim HJ, Kim YH, Park HS, Park NY (2004). Comparison of one-step RT-PCR and a nested PCR for the detection of canine distemper virus in clinical samples. Aust Vet J.

[8] Elia G, Decaro N, Martella V, Cirone F, Lucente MS, Lorusso E, Di Trani L, Buonavoglia C (2006). Detection of canine distemper virus in dogs by real-time RT-PCR. J Virol Methods.

[9] Cho HS, Park NY (2005). Detection of canine distemper virus in blood samples by reverse transcription loop-mediated isothermal amplification. J Vet Med B Infect Dis Vet Public Health.

[10] Piepenburg O, Williams CH, Stemple DL, Armes NA (2006). DNA detection using recombination proteins. PLoS Biol.

[11] Daher RK, Stewart G, Boissinot M, Bergeron MG (2016). Recombinase polymerase amplification for diagnostic applications. Clin Chem.

[12] Daher RK, Stewart G, Boissinot M, Boudreau DK, Bergeron MG (2015). Influence of sequence mismatches on the specificity of recombinase polymerase amplification technology. Mol Cell Probes.

[13] Abd El Wahed A, El-Deeb A, El-Tholoth M, Abd El Kader H, Ahmed A, Hassan S, Hoffmann B, Haas B, Shalaby MA, Hufert FT (2013). A portable reverse transcription recombinase polymerase amplification assay for rapid detection of foot-and-mouth disease virus. PLoS One.

[14] Boyle DS, Lehman DA, Lillis L, Peterson D, Singhal M, Armes N, Parker M, Piepenburg O, Overbaugh J. Rapid detection of HIV-1 proviral DNA for early infant diagnosis using recombinase polymerase amplification. MBio. 2013;4(2) doi:https://doi.org/10.1128/mBio.00135-13. 10.1128/mBio.00135-13 PMC3622927 23549916

[15] Crannell ZA, Cabada MM, Castellanos-Gonzalez A, Irani A, White AC, Richards-Kortum R (2015). Recombinase polymerase amplification-based assay to diagnose Giardia in stool samples. Am J Trop Med Hyg.

[16] Yang Y, Qin X, Zhang W, Li Y, Zhang Z (2016). Rapid and specific detection of porcine parvovirus by isothermal recombinase polymerase amplification assays. Mol Cell Probes.

[17] Boyle DS, McNerney R, Teng Low H, Leader BT, Perez-Osorio AC, Meyer JC, O'Sullivan DM, Brooks DG, Piepenburg O, Forrest MS (2014). Rapid detection of mycobacterium tuberculosis by recombinase polymerase amplification. PLoS One.

[18] Wang J, Liu L, Li R, Yuan W (2016). Rapid detection of porcine circovirus 2 by recombinase polymerase amplification. J Vet Diagn Investig.

[19] Murinda SE, Ibekwe AM, Zulkaffly S, Cruz A, Park S, Razak N, Paudzai FM, Ab Samad L, Baquir K, Muthaiyah K (2014). Real-time isothermal detection of Shiga toxin-producing Escherichia Coli using recombinase polymerase amplification. Foodborne Pathog Dis.

[20] Zaghloul H, El-Shahat M (2014). Recombinase polymerase amplification as a promising tool in hepatitis C virus diagnosis. World J Hepatol.

[21] Krolov K, Frolova J, Tudoran O, Suhorutsenko J, Lehto T, Sibul H, Mager I, Laanpere M, Tulp I, Langel U (2014). Sensitive and rapid detection of Chlamydia trachomatis by recombinase polymerase amplification directly from urine samples. J Mol Diagn.

[22] Yang D, Lv P, Hu C, Jia Y, Xie Z, Cao D, Ren Y, Liu H (2008). Isolation and identification of canine distemper virus from fox. Southwest China Journal of Agricultural Science.

[23] Yun JJ, Heisler LE, Hwang II, Wilkins O, Lau SK, Hyrcza M, Jayabalasingham B, Jin J, McLaurin J, Tsao MS (2006). Genomic DNA functions as a universal external standard in quantitative real-time PCR. Nucleic Acids Res.

[24] Lillis L, Siverson J, Lee A, Cantera J, Parker M, Piepenburg O, Lehman DA, Boyle DS (2016). Factors influencing Recombinase polymerase amplification (RPA) assay outcomes at point of care. Mol Cell Probes.

